# Dyspnea on Exercise Is Associated with Overall Symptom Burden in Patients with Chronic Respiratory Insufficiency

**DOI:** 10.1089/pmr.2020.0112

**Published:** 2021-03-02

**Authors:** Heidi A. Rantala, Sirpa Leivo-Korpela, Juho T. Lehto, Lauri Lehtimäki

**Affiliations:** ^1^ Department of Respiratory Medicine, Tampere University Hospital, Tampere, Finland.; ^2^ Faculty of Medicine and Health Technology, Tampere University, Tampere, Finland.; ^3^ Department of Oncology, Palliative Care Centre, Tampere University Hospital, Tampere, Finland.; ^4^ Allergy Centre, Tampere University Hospital, Tampere, Finland.

**Keywords:** chronic obstructive pulmonary disease, chronic respiratory insufficiency, dyspnea on exercise, Edmonton Symptom Assessment System, interstitial lung disease, modified Medical Research Council dyspnea questionnaire

## Abstract

***Background:*** Patients with chronic respiratory insufficiency suffer from many symptoms together with dyspnea.

***Objective:*** We evaluated the association of dyspnea on exercise with other symptoms in patients with chronic respiratory insufficiency due to chronic obstructive pulmonary disease or interstitial lung disease.

***Design:*** This retrospective study included 101 patients in Tampere University Hospital, Finland. Dyspnea on exercise was assessed with modified Medical Research Council (mMRC) dyspnea questionnaire, and other symptoms were assessed with Edmonton Symptom Assessment System (ESAS) and Depression Scale (DEPS). The study was approved by Regional Ethics Committee of Tampere University Hospital, Finland (approval code R15180/December 1, 2015).

***Results:*** Patients with mMRC 4 (most severe dyspnea) compared with those with mMRC 0–3 reported higher symptom scores on ESAS in shortness of breath (median 8.0 [IQR 6.0–9.0] vs. 4.0 [2.0–6.0], *p* < 0.001), dry mouth (7.0 [4.0–8.0] vs. 3.0 [1.0–6.0], *p* < 0.001), tiredness (6.0 [3.0–7.0] vs. 3.0 [1.0–5.0], *p* < 0.001), loss of appetite (3.0 [0.0–6.0] vs. 1.0 [0.0–3.0], *p* = 0.001), insomnia (3.0 [1.0–7.0] vs. 2.0 [0.0–3.0], *p* = 0.027), anxiety (3.0 [0.0–5.5] vs. 1.0 [0.0–3.0], *p* = 0.007), and nausea (0.0 [0.0–2.0] vs. 0.0 [0.0–0.3], *p* = 0.027). Patients with mMRC 4 were more likely to reach the DEPS threshold for depression than those scoring mMRC 0–3 (42.1% vs. 20.8%, *p* = 0.028).

***Conclusions:*** Patients with chronic respiratory insufficiency need comprehensive symptom screening with relevant treatment, as they suffer from broad symptom burden worsening with increased dyspnea on exercise.

## Introduction

Vast majority of patients with chronic obstructive pulmonary disease (COPD) or interstitial lung disease (ILD) suffer from dyspnea, but only a smaller fraction with advanced disease have chronic respiratory insufficiency, a marker of impaired life expectancy. Dyspnea, which may occur with or without respiratory insufficiency, is a subjective experience of breathing discomfort,^1^ whereas chronic respiratory insufficiency is an objective finding defined by hypoxemia (partial pressure of oxygen in arterial gas <8.0 kPa) or hypercapnia (partial pressure of carbon dioxide in blood gas ≥6.0 kPa) caused by disturbance of gas exchange between pulmonary alveoli and circulation or by insufficient ventilation.^2^

Patients with advanced COPD or ILD typically suffer from severe dyspnea,^3–5^ which increases with approaching death and is associated with impaired quality of life.^6–8^ In addition to dyspnea, patients with COPD and ILD suffer frequently from other symptoms, such as fatigue, weight loss, depression, and anxiety, further impairing their quality of life.^9^ Previous studies have shown the association of dyspnea and other symptoms in patients with COPD and ILD in general.^3,10,11^ However, the overall symptom burden specifically in patients with chronic respiratory insufficiency due to COPD or ILD and increasing dyspnea remains unknown.

Centers managing patients with chronic respiratory insufficiency commonly screen for dyspnea as a target of therapy. However, assessment of other symptoms and their association with increasing dyspnea would be important to offer more comprehensive treatment for these patients.

Our aim was to assess how dyspnea on exercise is associated with overall symptom burden in patients with chronic respiratory insufficiency due to COPD or ILD.

## Materials and Methods

This was a retrospective study performed in patients with chronic respiratory insufficiency visiting the respiratory insufficiency clinic of Tampere University Hospital between 1.10.2016 and 31.10.2017. All the patients with chronic respiratory insufficiency due to COPD or ILD, who had filled in the modified Medical Research Council (mMRC) dyspnea questionnaire during the routine visits, were included. Patients' clinical characteristics, diagnoses, mMRC dyspnea scores, Edmonton Symptom Assessment System (ESAS), and Depression Scale (DEPS) were collected from medical records. The Charlson comorbidity index (CCI) was calculated for each patient.^12,13^

### Questionnaires

The mMRC questionnaire asks patients to self-report dyspnea in daily activities. The scale varies from 0 to 4: 0 for “I only get breathless with strenuous exercise,” 1 for “I get short of breath when hurrying on the level or up a slight hill,” 2 for “I walk slower than people of the same age on the level because of breathlessness or have to stop for breath when walking at my own pace on the level,” 3 for “I stop for breath after walking about 100 meters or after a few minutes on the level,” and 4 for “I am too breathless to leave the house.”^14,15^

ESAS is used for assessing symptoms in many advanced diseases.^16,17^ Patients rate different symptoms on a numeric rating scale from 0 (no symptoms) to 10 (the worst possible symptoms).^18,19^ We used a modified version with 12 questions covering 11 symptoms and general well-being (0 for the best possible well-being and 10 for the worst possible well-being). The cutoff point for each symptom to be categorized as moderate or severe was ≥4.^20–22^

The DEPS is a validated self-assessed screening tool for depression consisting of 10 questions and provides a total score varying from 1 to 30 points.^23^ The suggested cutoffs for depressive symptoms and clinical depression are ≥9 and ≥12, respectively.^24^

### Statistical analysis

The five-step mMRC scale was converted to two-step scale by comparing scores 0–3 and 4 to sort out the group with most difficult dyspnea on exercise. Comparisons of different groups were performed by Mann-Whitney *U* test for continuous variables as the distributions were non-normal based on visual estimation, and Pearson's chi-square or Fisher's exact tests for categorical variables.

To assess if the relation between dyspnea on exercise and other symptoms is independent of other clinical factors, we conducted a logistic regression multivariate analysis including also gender, age, body mass index, primary diagnosis for chronic respiratory insufficiency, CCI, and ESAS total score. Statistical significance was set as *p* < 0.05. Analyses were performed with IBM SPSS Statistics version 26.0. (IBM Corp, Armonk, NY).

### Ethics approval and consent to participate

This study was approved by the Regional Ethics Committee of Tampere University Hospital, Finland (approval code R15180/December 1, 2015).

## Results

During the follow-up time, 128 patients with COPD or ILD and chronic respiratory insufficiency visited the clinic. The mMRC questionnaire was available in 101 patients, among whom ESAS and DEPS questionnaires were available in 98 and 91 patients, respectively. Reasons for the missing ESAS or DEPS questionnaires were unwillingness to answer the questionnaire, inability to complete the questionnaire, and technical or unknown reasons.

The patient characteristics are shown in Table 1. COPD was severe (GOLD grade III: FEV_1_ 30%–50% predicted) or very severe (GOLD grade IV: FEV_1_ <30% predicted)^9^ in most (75.2%) of the patients with COPD. Patients in mMRC category 4 were more likely to need help in activities of daily living and had lower FEV_1_ and body mass index than those scoring 0–3 in mMRC. The treatment for respiratory insufficiency was oxygen therapy in 81 (80.2%), noninvasive ventilation (NIV) in 10 (9.9%), and both in 6 (5.9%) patients. Four patients (4.0%) refused to use NIV or oxygen therapy despite chronic respiratory insufficiency. Of the deceased patients, 29 (60.4%) died during the following year after the visit in the clinic.

**Table 1.** Patient Characteristics According to the Modified Medical Research Council Dyspnea Scale Category

	All patients	mMRC 0–3	mMRC 4	p^a^
Total, *n*	101	55	46	
Male, *n* (%)	65 (64.4)	36 (65.5)	29 (63.0)	0.801
Age, years, median (IQR)	75.0 (70.0–81.0)	74.0 (69.0–80.0)	75.5 (71.0–81.5)	0.141
BMI, kg/m^2^, median (IQR)	24.5 (21.1–29.3)	27.0 (22.5–33.2)	23.3 (19.3–27.7)	0.001
Smoking status, *n* (%)				
Never-smoker	9 (8.9)	3 (5.5)	6 (13.0)	0.104
Ex-smoker	89 (88.1)	48 (87.3)	40 (87.0)	
Smoker	4 (4.0)	4 (7.3)	0 (0.0)	
Disease causing the chronic respiratory insufficiency, *n* (%)				
COPD	89 (88.1)	47 (85.5)	42 (91.3)	0.366
ILD	12 (11.9)	8 (14.5)	4 (8.7)	
FEV_1_				
Liters, median (IQR)	0.90 (0.60–1.25)	0.98 (0.68–1.48)	0.72 (0.46–1.1)	0.008
% of predicted, median (IQR)	31.0 (23.0–48.5)	34.0 (27.0–54.0)	25.5 (19.0–44.3)	0.003
Charlson comorbidity index, median (IQR)	2.0 (0.0–2.0)	2.0 (0.0–2.0)	2.0 (0.0–2.0)	0.789
Need for help with ADL, *n* (%)	38 (37.6)	9 (16.4)	29 (63.0)	<0.001
Died before 31.12.2018, *n* (%)	48 (47.5)	22 (40.0)	26 (56.5)	0.098

^a^ Between the patients in categories mMRC 0–3 and mMRC 4.

ADL, activities of daily living; BMI, body mass index; COPD, chronic obstructive pulmonary disease; FEV_1_, forced expiratory volume in one second; ILD, interstitial lung disease; IQR, interquartile range; mMRC, modified Medical Research Council.

The symptom severities measured by ESAS in the two mMRC categories are shown in Table 2. In the total study population, shortness of breath and dry mouth were the most severe symptoms. Compared with patients with mMRC 0–3, those with mMRC 4 reported significantly higher scores in shortness of breath, dry mouth, tiredness, loss of appetite, anxiety, insomnia, nausea, and impaired well-being.

**Table 2.** Median Scores and Proportion of Patients with at Least Moderate Symptoms (≥4 Points) in Edmonton Symptom Assessment System Questionnaire According to Modified Medical Research Council Dyspnea Scale Category

	All (n = 98)	mMRC 0–3 (n = 55)	mMRC 4 (n = 43)	p^a^
ESAS scores^b^				
Pain at rest				
Median (IQR)	0.0 (0.0–3.0)	0.0 (0.0–3.0)	2.0 (0.0–4.0)	0.063
≥4, %	21.9	17.0	27.9	0.198
Pain on movement				
Median (IQR)	2.0 (0.0–6.0)	2.0 (0.0–4.0)	5.0 (0.0–6.0)	0.068
≥4, %	41.7	30.2	55.8	0.011
Tiredness				
Median (IQR)	3.0 (2.0–6.0)	3.0 (1.0–5.0)	6.0 (3.0–7.0)	<0.001
≥4, %	49.0	32.1	69.8	<0.001
Shortness of breath				
Median (IQR)	6.0 (3.0–8.0)	4.0 (2.0–6.0)	8.0 (6.0–9.0)	<0.001
≥4, %	72.2	57.4	90.7	<0.001
Loss of appetite				
Median (IQR)	1.0 (0.0–5.0)	1.0 (0.0–3.0)	3.0 (0.0–6.0)	0.001
≥4, %	28.9	14.8	46.5	0.001
Nausea				
Median (IQR)	0.0 (0.0–1.0)	0.0 (0.0–0.3)	0.0 (0.0–2.0)	0.027
≥4, %	6.2	3.7	9.3	0.255
Dry mouth				
Median (IQR)	5.0 (2.0–7.0)	3.0 (1.0–6.0)	7.0 (4.0–8.0)	<0.001
≥4, %	60.2	47.3	76.7	0.003
Constipation				
Median (IQR)	1.0 (0.0–4.0)	1.0 (0.0–3.0)	2.0 (0.0–6.0)	0.072
≥4, %	28.9	18.5	41.9	0.012
Depression				
Median (IQR)	1.0 (0.0–4.0)	1.0 (0.0–3.0)	2.0 (0.0–5.0)	0.120
≥4, %	30.2	22.2	40.5	0.053
Anxiety				
Median (IQR)	1.0 (0.0–4.8)	1.0 (0.0–3.0)	3.0 (0.0–5.5)	0.007
≥4, %	32.3	22.2	45.2	0.017
Insomnia				
Median (IQR)	2.0 (0.0–4.0)	2.0 (0.0–3.0)	3.0 (1.0–7.0)	0.027
≥4, %	28.1	18.9	39.5	0.025
Well-being				
Median (IQR)	4.0 (3.0–5.0)	3.0 (2.0–5.0)	5.0 (4.0–6.0)	<0.001
Total score				
Median (IQR)	34.0 (21.0–51.5)	24.0 (15.8–34.8)	44.0 (34.0–63.0)	<0.001

^a^ Between the patients in categories mMRC 0–3 and mMRC 4.

^b^ Data missing in three patients: *inability to fill in the questionnaire (2), unwillingness to answer the questionnaire (1)*.

ESAS, Edmonton Symptom Assessment System.

A significantly higher proportion of patients with mMRC 4, compared with those scoring 0–3 in mMRC, reached the threshold for moderate or severe symptom (≥4) in shortness of breath, pain on movement, tiredness, loss of appetite, constipation, anxiety, insomnia, and dry mouth. The total ESAS score among patients in mMRC category 4 compared with those in mMRC 0–3 category remained statistically significantly higher also in the logistic regression multivariate analysis accounting for other clinical factors.

The scores of DEPS in the two mMRC categories are shown in Table 3. As compared with patients scoring 0–3 in mMRC, those in mMRC category 4 had higher median DEPS scores and a significantly higher proportion of them reached the threshold for clinical depression.

**Table 3.** Median Scores and Proportion of Patients with at Least 9 or 12 Points in Depression Scale Questionnaire According to Modified Medical Research Council Dyspnea Scale Category

	All (n = 91)	mMRC 0–3 (n = 53)	mMRC 4 (n = 38)	p^a^
DEPS score, median (IQR)^b^	8.0 (3.0–14.0)	6.0 (2.5–10.5)	9.5 (4.8–18.5)	0.025
DEPS ≥9 points, *n* (%)	41 (40.6)	20 (37.7)	21 (55.3)	0.097
DEPS ≥12 points, *n* (%)	27 (26.7)	11 (20.8)	16 (42.1)	0.028

^a^ Between patients in categories mMRC 0–3 and mMRC 4.

^b^ Data missing in 10 patients: *inability to complete in the questionnaire (2), unwillingness to answer DEPS questionnaire (3), technical or unknown reason (5)*.

## Discussion

We identified a high symptom burden among patients with chronic respiratory insufficiency due to COPD or ILD. Patients with more severe dyspnea on exercise and scoring 4 in mMRC had more severe symptoms of dry mouth, tiredness, loss of appetite, anxiety, nausea, and insomnia in addition to impaired well-being measured with ESAS, compared with those with mMRC 0–3. Also depression measured with DEPS was more common in patients with mMRC 4 than in patients with mMRC 0–3.

Our finding that patients with COPD or ILD suffer from many symptoms, which worsens by increasing dyspnea on exercise, is in line with previous studies.^25,26^ However, although earlier studies have shown that symptoms are worse in those patients with COPD or ILD who suffer from more severe dyspnea,^3,27^ this is the first study to assess this specifically in patients with chronic respiratory insufficiency.

Many of the symptoms found in this study, such as fatigue, loss of appetite, and tiredness, may be consequences of an advanced disease.^28^ In contrast, some of the symptoms, for example, dry mouth, could be directly associated with dyspnea on exercise as a result of mouth breathing and higher frequency of breathing, but also oxygen therapy or NIV and used medication, for example, inhaled anticholinergics, may provoke dryness of mouth.

Scoring at least 12 points in DEPS questionnaire, the cutoff for depression,^24^ was significantly more common in patients with mMRC score 4 than in those with mMRC score 0–3. This is in line with previous studies that have focused on the same relation from the opposite perspective and showed higher levels of dyspnea and other symptoms in patients with COPD suffering from anxiety and depression.^29,30^ Dyspnea has also been associated with higher depression scores in patients with ILD.^10^ In a previous study on an unselected population of patients with chronic respiratory insufficiency, one third of the patients suffered from depressive symptoms and a quarter from depression,^31^ being less than in this study focusing only on patients with COPD or ILD causing respiratory insufficiency.

The patients with mMRC score 4 have, by definition, restricted ability to leave home or take part in activities, which may lead to social exclusion and depression. This further underlines the importance of screening depression in patients with chronic respiratory insufficiency to find those patients who will benefit from the treatment of depression.

### Strengths and limitations

This was a retrospective study performed in patients with chronic respiratory insufficiency due to COPD or ILD, offering practical information on symptom burden of these patients. Owing to the retrospective nature of the study, there were some questionnaire data missing. This may have biased the sample to those with less severe symptoms, and thereby underestimated the total symptom burden of the patient population. Medical treatment of the underlying pulmonary disease and treatment of chronic respiratory insufficiency may affect the relationship between dyspnea on exercise and other symptoms, but we were not able to assess this effect in our cross-sectional setting.

Further long-term follow-up studies would provide more information on how the relationship between dyspnea on exercise and other symptoms develop during the course of the disease.

## Conclusions

In patients with chronic respiratory insufficiency due to pulmonary disease increasing dyspnea on exercise is associated with higher overall symptom burden, especially symptoms such as dry mouth, tiredness, loss of appetite, anxiety, nausea, depression, and insomnia. Therefore, these patients need a comprehensive symptom screening and management, including psychosocial support and early integrated palliative care.

## Abbreviations Used

ADL: activities of daily living
BMI: body mass index
COPD: chronic obstructive pulmonary disease
CPAP: continuous positive airway pressure
DEPS: Depression Scale
ESAS: Edmonton Symptom Assessment System
FEV_1_: forced expiratory volume in one second
ILD: interstitial lung disease
IQR: interquartile range
mMRC: modified Medical Research Council
NIV: noninvasive ventilation
